# Treatment challenges of ruptured intracranial aneurysms during pregnancy: A case record and review of the literature

**DOI:** 10.1016/j.bas.2024.103911

**Published:** 2024-09-24

**Authors:** Christian Blume, Christian Mayer, Matthias Simon, Walid Albanna, Azize Boström

**Affiliations:** a Department of Neurosurgery, University of Aachen, Pauwelstrasse 30, 52074, Aachen, Germany; b Radiologisches Institut Dr. von Essen, Emil-Schüller-Strasse 33, 56068, Koblenz, Germany; c Department of Neurosurgery, University of Bielefeld Medical Center, Burgsteig 13, 33617, Bielefeld, Germany; d Institute for Neurophysiology, University of Cologne, Robert-Koch-Str. 39, 50931, Cologne, Germany; e Mediclin Robert Janker Clinic Bonn, Villenstraße 8, 53129, Bonn, Germany

**Keywords:** SAH, Pregnancy, Aneurysmal, Cerebral aneurysma

## Abstract

**Introduction:**

Aneurysmal subarachnoid hemorrhage (aSAH) during pregnancy is a rare but serious complication associated with significant maternal and fetal morbidity and mortality. Due to a limited number of published cases, development of guidelines for the management of aSAH in pregnant women has proven difficult. In the present article, we present a case and review the available literature on patients with aSAH during pregnancy.

**Research question:**

What is the optimal management of aSAH during pregnancy?

**Material and methods:**

We describe the case of a pregnant woman with aSAH. In addition, a search of the PUBMED database was conducted to collect all pertinent case reports of aSAH in pregnant women published since 1990.

**Results:**

A 36 years old Caucasian primigravid woman in the 37th GW presented to our department with aSAH, due to rupture of a saccular basilar tip aneurysm. After multidisciplinary discussion, a Caesarian section (CS) and subsequent aneurysm treatment by endovascular coiling were performed without complications. On day four after ictus endovascular spasmolysis were initiated as the patient developed angiographic cerebral vasospasm and delayed cerebral ischemia (DCI). Two days later, brain tissue hypoperfusion was further aggravated by cardiopulmonary failure under induced hypertension, so that the patient died on day seven from severe cerebral infarction.

**Discussion and conclusion:**

While there are still no formal studies that could guide the optimal management of aSAH during pregnancy, primary CS prior to definitive management of ruptured aneurysms during the third trimester seems to be the safest treatment approach for both mother and child.

## Highlights

• SAH during pregnancy has severe consequences for both mother and baby.
• We describe the case of a pregnant woman with aSAH.
• A search of the PUBMED database was conducted.
• We collected case series of aSAH in pregnant women published since 1990.

## 1 Introduction

Aneurysmal subarachnoid hemorrhage (aSAH) due to rupture of an intracranial aneurysm with extravasation of blood into the subarachnoid space is a relatively rare but serious form of stroke with complex pathophysiology that comprises both early and delayed mechanisms of brain damage (Kaptain et al., 2000; Weaver and Fisher, 1994; Geraghty and Testai, 2017). The exact incidence of aSAH during pregnancy remains a matter of debate, but it has been estimated to be in the order of 1–2 per 10.000 pregnancies and to be increased compared to the general population (Selo-Ojeme et al., 2004). Owing to the physiological changes associated with pregnancy and the potentially severe consequences for both mother and baby, treatment of aSAH in pregnant women remains a particularly challenging and multidisciplinary task. Treatment decisions in these patients are further complicated by the limited amount of literature on the topic, which consists mainly of case reports and small case series, and the resulting lack of reliable data. Thus, despite improved treatment approaches, maternal morbidity and mortality in pregnant women suffering from aSAH remain significant (Dias and Sekhar, 1990), as reflected in the fact that aSAH represents one of the leading causes of indirect maternal death (Selo-Ojeme et al., 2004; D'Haese et al., 1997). The aim of the present case report and subsequent review of the relevant literature is to offer guidance for neurosurgeons as well as physicians from other disciplines involved in the treatment of this uncommon group of patients.

## 2 Methods

We describe the case of a pregnant woman in the 37th gestational week (GW) who presented with aSAH due to rupture of a basilar artery aneurysm and was treated in our department. For the literature review, we conducted a PUBMED database search with the terms “pregnancy”, “aneurysmal subarachnoid hemorrhage”, “endovascular embolization”, and “cerebral aneurysm” to collect all pertinent articles published on the topic since 1990. All case reports and case series of pregnant women with aSAH were selected and analyzed in terms of patient age, gestational week, aneurysm location, mode of delivery, aneurysm treatment, chronology of delivery and treatment, as well as maternal and fetal outcome.

## 3 Results

### 3.1 Case illustration

**Fig. 1:**
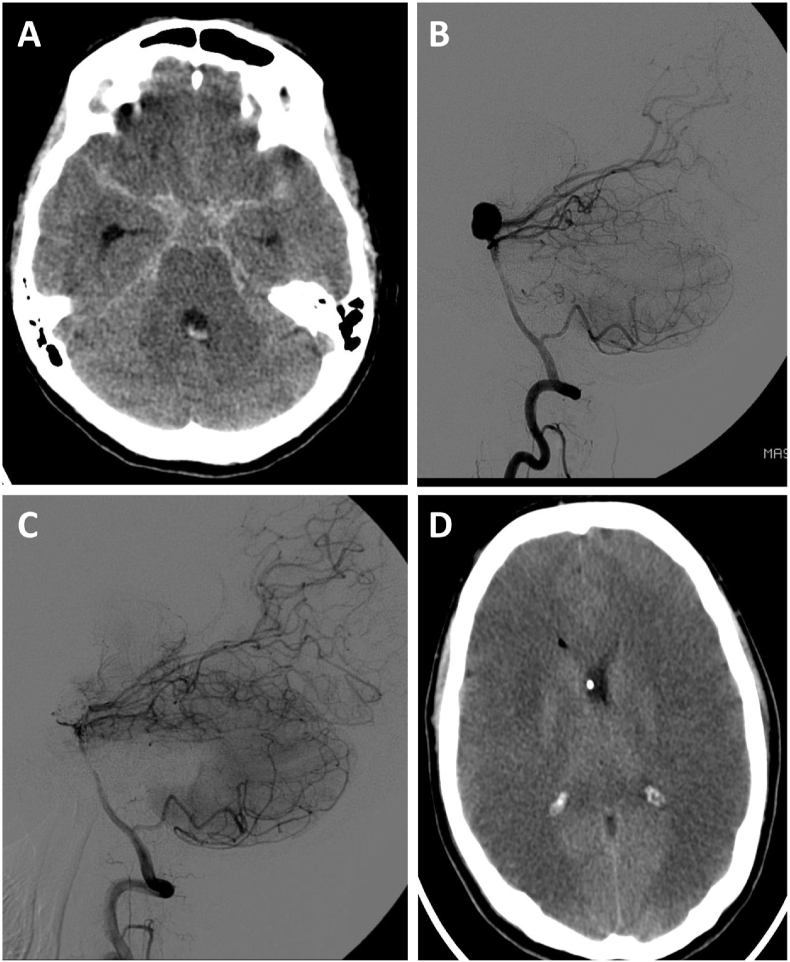
**Imaging data from the patient described in our case report of aSAH during pregnancy.** (**A**) Initial CT scan showing symmetric subarachnoid hemorrhage around the circle of Willis and the basal cistern as well as associated hydrocephalus. (**B & C**) Digital subtraction angiography (DSA) showing a 11 × 9 mm saccular basilar tip aneurysm before (**B**) and after (**C**) coil embolization of the aneurysm. (**D**) CT scan showing bi-hemispheric infarction of the middle cerebral artery (MCA) and anterior cerebral artery (ACA) areas as well as an associated bi-hemispheric cerebral edema with compression of the ventricular system.

A 36 years old Caucasian primigravid woman in the 37th GW presented to our department with aSAH, World Federation of Neurosurgical Societies (WFNS) Grade II, due to rupture of a saccular basilar tip aneurysm (Fig. 1A and **B**). Following a multidisciplinary discussion with obstetrician, neuroradiologist, anaesthesiologist, neurosurgeon and paediatrist, a Caesarean section (CS) was performed before treatment of the ruptured aneurysm. Based on its location and nature, the ruptured aneurysm was subsequently treated by endovascular coil embolization (Fig. 1C). Both procedures could be performed without any complications, and neither mother nor child showed any immediate deficits.

However, four days after ictus, the patient became increasingly confused and digital subtraction angiography (DSA) showed signs of cerebral vasospasm, so that induced hypertension with catecholamines was initiated. Following another DSA that revealed obvious vasospasm, intermittent endovascular spasmolysis with nimodipine was initiated. On day six after ictus, the patient developed severe cardiopulmonary failure under induced hypertension with catecholamines, so that the target middle arterial pressure (MAP) of 100 mmHg could no longer be maintained. Despite ultima ratio treatment by intra-aortic balloon counter-pulsation (IABP), the patient died on day seven from severe bi-hemispheric cerebral infarction (Fig. 1D).

### 3.2 Literature review

**Fig. 2:**
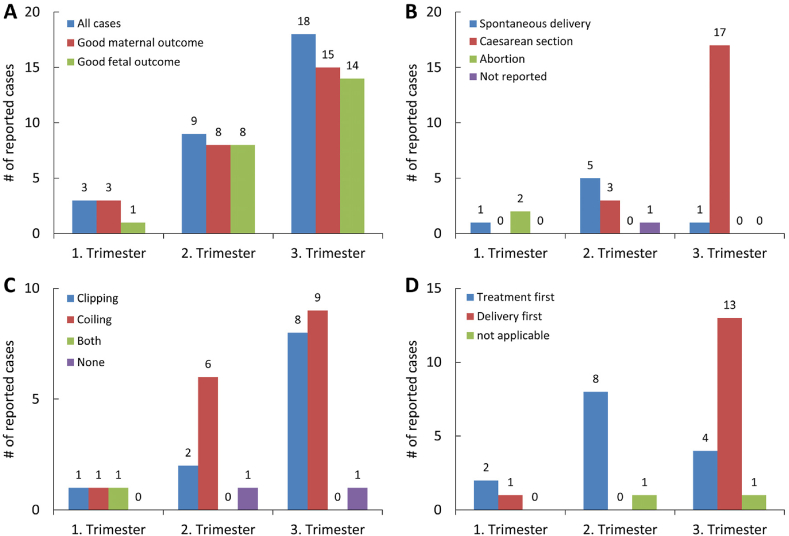
Summary of (**A**) maternal and fetal outcomes, (**B**) obstetric procedures, (**C**) treatment modalities and (**D**) chronology of treatment/delivery in 30 previously reported cases of aSAH during different stages of pregnancy. For details see Table 1.

A total of 30 previously reported cases of aSAH in pregnant women (age: 19–41 years; GW: 10th-39th week) were identified in our literature review (Table 1). The aneurysms were most frequently located in the internal carotid artery (10/30 or 33%), followed by the anterior communicating artery (5/30 or 17%), the posterior communicating, basilar or posterior cerebral artery (3/30 or 10% each), the posterior inferior cerebellar or middle cerebral artery (2/30 or 7% each) and the anterior cerebral artery (1/30 or 3%) (Table 2). Overall, two of the patients (7%) died before the aneurysm could be treated, although in one of these cases, the child could be rescued by a CS. Of the remaining patients, 11 (37%) were treated by microsurgical clipping, 16 (53%) by endovascular coiling and one (3%) by successive microsurgical clipping and endovascular coiling of the ruptured aneurysm. Maternal outcome was good (no reported impairments or score of 4–5 on the Glasgow outcome scale) in all 11 patients treated by microscurgical clipping only, in 14 of the 16 (87.5%) patients treated by endovascular coiling only as well as in the one patient treated with microsurgical clipping and endovascular coiling (Table 2). The two remaining patients treated by endovascular coiling died. Thus, when considering all reported cases, maternal outcome was good in 26 (87%) of the patients and poor (death) in 4 (13%) of the patients (Table 2). Fetal outcome was good (no signs of impairment) in 23 (77%) of the cases, poor (death) in three (10%) of the cases and not reported in four (13%) of the cases (Table 2). With regard to the stage of pregnancy, three (10%) patients presented during the first trimester, nine (30%) during the second trimester and 18 (60%) during the third trimester (Fig. 2A). Of the patients who presented during the first trimester, two had an induced abortion (Fig. 2B), one patient before endovascular coil embolization of the aneurysm and the second after microsurgical clipping. The third patient was treated by microsurgical clipping, continued pregnancy and delivered a healthy baby spontaneously. Maternal outcome was good in all three patients who presented during the first trimester (Fig. 2A). In the patients who presented during the second trimester, no induced abortion was performed (Fig. 2B). Except for one patient who died before treatment, all aneurysms in this group were successfully treated (six by coiling and two by clipping, Fig. 2C) and the pregnancies were continued in all patients. Five patients ranging from 16th to 28th GW delivered their children spontaneously and three patients ranging from 16th or 28th GW had a CS, respectively (Fig. 2B). The children were all healthy and showed no deficits or abnormalities. Of the patients who presented during the third trimester, one patient died before treatment while nine were treated by endovascular coil embolization and eight by microsurgical clipping (Fig. 2C). Except for one patient who was treated by endovascular coiling and delivered her child spontaneously, all patients who presented during the 3rd trimester had a CS (13 before and four after treatment, Fig. 2D). Maternal outcome was good in all but two of these patients, who died following delivery by a CS and subsequent coiling of the aneurysm. When considering all reported cases regardless of GW, 14 patients (47%) were treated before delivery, 13 patients (43%) were treated after delivery and one patient (3%) was treated after induced abortion. Maternal outcome was good in all 14 patients treated before delivery and in the patient treated after induced abortion, while two of the 13 patients (15%) treated after delivery died.

**Table 1:** Overview of our own and 30 previously reported cases of aSAH during pregnancy.

Ref.	Age	Aneurysm location	GW	Obstetric procedure	Aneurysm treatment	Chronology	Outcome (fetal/maternal^b^)
a	36	BA	37	CS	Coiling	Delivery first	good/dead
Whitburn et al. (1990)	38	AcomA	35	CS	Clipping	Delivery first	good/good
El Gawly (1992)	31	ICA	39	CS	Clipping	Delivery first	good/good
Kriplani et al. (1995)	25	AcomA	37	CS	Clipping	Treatment first	good/good
Kriplani et al. (1995)	26	PICA	36	CS	Clipping	Delivery first	good/good
D'Haese et al., 1997	29	ICA	34	CS	Clipping	Delivery first	good/good
Meyers et al. (2000)		PCA	11	SD	Clipping + Coiling	Treatment first	good/good
Meyers et al. (2000)	36	BA	32	SD	Coiling	Treatment first	good/good
Meyers et al. (2000)	36	PcomA	36	CS	Coiling	Delivery first	good/good
Jaeger et al. (2000)	38	ICA	36	CS	Clipping	Delivery first	good/good
Piotin et al. (2001)	28	ICA	32	CS	Coiling	Delivery first	good/good
Piotin et al. (2001)	31	ICA	22	SD	Coiling	Treatment first	good/good
Shahabi et al. (2001)	36	BA	38	CS	Coiling	Delivery first	good/good
Kizilkilic et al. (2003)	25	PcomA	10	Abortion	Coiling	Treatment first	dead/ good
Kizilkilic et al. (2003)	39	ICA	18	SD	Coiling	Treatment first	good/good
Kizilkilic et al. (2003)	26	AcomA	28	SD	Coiling	Treatment first	good/good
Nelson (2005)	19	AcomA	16	SD	Clipping	Treatment first	good/good
Tiel Groenestege et al. (2009)	32	ICA	10	Abortion	Clipping	Abortion first	dead/good
Tiel Groenestege et al. (2009)	23	ICA	17	CS	Clipping	Treatment first	good/good
Tiel Groenestege et al. (2009)	31	unknown	28	unknown	none	–	dead/dead
Tiel Groenestege et al. (2009)	32	PCA	38	CS	none	–	good/ dead
Elwatidy et al. (2011)	26	ICA	36	CS	Clipping	Delivery first	good/good
Elwatidy et al. (2011)	24	MCA	22	SD	Coiling	Treatment first	good/good
Tarnaris et al. (2012)	21	PcomA	29	CS	Coiling	Treatment first	good/good
Guida et al. (2012)	37	ACA	34	CS	Clipping	Delivery first	unknown/good
Guida et al. (2012)	34	MCA	37	CS	Coiling	Delivery first	unknown/dead
Guida et al. (2012)	41	AcomA	32	CS	Coiling	Delivery first	unknown/dead
Guida et al. (2012)	28	BA	33	CS	Coiling	Delivery first	unknown/good
Vatsa et al. (2019)	25	PCA	28	CS	Coiling	Treatment first	good/good
Xie et al. (2021)	28	ICA	32	CS	Coiling	Treatment first	good/good
Kim et al. (2014)	34	PICA	16	CS	Coiling	Treatment first	good/good

Abbreviations: ACA, anterior cerebral artery; AcomA, anterior communicating artery; BA, basilar artery; CS, caesarean section; GOS, Glasgow outcome scale; GW, gestational week; ICA, internal carotid artery; MCA, middle cerebral artery; PCA, posterior cerebral artery; PComA, posterior communicating artery; PICA, posterior inferior cerebellar artery; SD, spontaneous delivery.

a Present case report.

b Good maternal outcome = no deficits or GOS 4–5.

**Table 2:** Summary of the patient characteristics for 30 previously reported cases of aSAH during pregnancy

Patient characteristic |n (%)	All	Good maternal outcome^a^	Poor maternal outcome^b^
**All cases**	30 (100%)	26/30 (87%)	4/30 (13%)
**Age**			
Median [q1-q3]	31 [26–36]	29 [25–36]	33 [32–36]
**Gestational week**			
Median [q1-q3]	32 [22–36]	32 [19–36]	35 [31–37]
1st trimester (1–12 wks)	3 (10%)	3/3 (100%)	0/3 (0%)
2nd trimester (13–28 wks)	9 (30%)	8/9 (89%)	1/9 (11%)
3rd trimester (29–40 wks)	18 (60%)	15/18 (83%)	3/18 (17%)
**Aneurysm location**			
Internal carotid artery (ICA)	10 (33%)	10/10 (100%)	0/10 (0%)
Anterior communicating artery (AComA)	5 (17%)	4/5 (80%)	1/5 (20%)
Posterior communicating artery (PComA)	3 (10%)	3/3 (100%)	0/3 (0%)
Basilar artery (BA)	3 (10%)	3/3 (100%)	0/3 (0%)
Posterior cerebral artery (PCA)	3 (10%)	2/3 (67%)	1/3 (33%)
Posterior inferior cerebellar artery (PICA)	2 (7%)	2/2 (100%)	0/2 (0%)
Middle cerebral artery (MCA)	2 (7%)	1/2 (50%)	1/2 (50%)
Anterior cerebral artery (ACA)	1 (3%)	1/1 (100%)	0/1 (0%)
Unknown	1 (3%)	0/1 (0%)	1/1 (100%)
**Aneurysm treatment**			
Microsurgical clipping	11 (37%)	11/11 (100%)	0/11 (0%)
Endovascular coiling	16 (53%)	14/16 (87.5%)	2/16 (12.5%)
Clipping + coiling	1 (3%)	1/1 (100%)	0/1 (0%)
None	2 (7%)	0/2 (0%)	2/2 (100%)
**Obstetric procedure**			
Spontaneous delivery (SD)	7 (23%)	7/7 (100%)	0/7 (0%)
Caesarean section (CS)	20 (67%)	17/20 (85%)	3/20 (15%)
Abortion	2 (7%)	2/2 (100%)	0/2 (0%)
Not reported	1 (3%)	0/1 (0%)	1/1 (100%)
**Chronology**			
Delivery first	13 (43%)	11/13 (85%)	2/13 (15%)
Treatment first	14 (47%)	14/14 (100%)	0/14 (0%)
Abortion first	1 (3%)	1/1 (100%)	0/1 (0%)
Not applicable	2 (7%)	0/2 (0%)	2/2 (100%)
**Fetal outcome**			
Good	23 (77%)	22/23 (96%)	1/23 (4%)
Death	3 (10%)	2/3 (67%)	1/3 (33%)
Not reported	4 (13%)	2/4 (50%)	2/4 (50%)

a Good maternal outcome = no deficits or GOS 4–5.

b Poor maternal outcome = death.

## 4 Discussion

aSAH during pregnancy is a rare but serious complication associated with significant risk for harm to both mother and child. Based on our review of 30 reported cases, aSAH in pregnant woman was associated with a maternal mortality of 13% and a fetal mortality of at least 10%, which corresponds well with previous estimates of 13–35% maternal and 7–25% fetal mortality (Dias and Sekhar, 1990; Amias, 1970; HISLEY et al., 1975; Robinson et al., 1972). Due to a lack of reliable data, the role of pregnancy as a potential risk factor for aSAH remains controversial. However, there are a number of plausible factors such as increased plasma volumes, higher rates of hypertension and other hemodynamic changes associated with pregnancy that could increase the risk of aSAH in pregnant woman (Selo-Ojeme et al., 2004; de la Monte et al., 1985). In addition, changes in the organization and content of arterial and venous vessel walls mediated by increased levels of progesterone, estrogen, relaxin and other hormones may predispose pregnant woman to the formation, rapid growth and rupture of cerebral aneurysms (de la Monte et al., 1985; BARRETT et al., 1982; Weir and Drake, 1991; Stehbens, 1981). Thus, the rate of growth of intracranial aneurysms and the risk of rupture have typically been reported to parallel the hemodynamic and hormonal changes associated with pregnancy and to increase towards the third trimester (Dias and Sekhar, 1990; Weir and Drake, 1991; Hunt et al., 1974; Desai et al., 2019). This is in line with the result of our literature review that aSAH in pregnant woman occurs most frequently during the third trimester (60%), followed by the second trimester (30%) and the third trimester (10%).

Even though the physiological changes associated with pregnancy as well as the potential risk to the fetus may complicate the management of aSAH in pregnant patients, it is generally accepted that prompt neurosurgical treatment should take precedence over obstetric considerations (Selo-Ojeme et al., 2004). As such, neither radiological nor surgical procedures should be avoided or delayed to protect the fetus, as the potential for harm is almost invariably outweighed by the potential benefit to both, mother and fetus. For example, radiation levels due to diagnostic procedures like X-ray or CT are typically far below the levels required to threaten the well-being of the fetus, especially if precautions such as fetal shielding are used to further reduce radiation exposure of mother and child (Brent, 1999; Hall, 1991; Marshman et al., 2005, 2007). Likewise, based on our review of the available literature, in all cases of aSAH during the second trimester, except for one patient who died before treatment, pregnancy could be continued after aneurysm treatment with good outcome for both mother and child. The reviewed cases also indicate that during the third trimester, development of the child has usually progressed far enough that CS before aneurysm treatment is a feasible option. In such cases, delivery by CS may not only reduce the potential for harm to the fetus, but also simplify subsequent aneurysm treatment as well as the management of complications. Thus, in the patient described in our own case report, CS with subsequent aneurysm treatment by endovascular coiling could be performed successfully and neither mother nor child showed immediate deficits. In the following days, the mother developed angiographic vasospasm and delayed cerebral ischemia (DCI), necessitating several rescue procedures (induced hypertension, intermittent endovascular spasmolysis) that might have been complicated by obstetric considerations if the pregnancy would have been continued. In the end, all treatment efforts were unsuccessful and brain tissue hypoperfusion was further aggravated by cardiopulmonary failure, so that the mother died on day seven due to severe cerebral infarction despite ultima ratio treatment by IABP. With regard to alternative treatment options, transcutaneous balloon angioplasty would most likely have provided little additional benefit in our patient, given that its effects are usually restricted to the treated, proximal vessel segments, with limited efficiency against distal vasoconstriction. On the other hand, continuous spasmolysis with intraarterial nimodipine has been shown to be an effective treatment in patients with sustained global vasospasm, especially when combined with multimodal neuromonitoring for continuous assessment of patient status and treatment efficiency (Weiss et al., 2019; Veldeman et al., 2020). Likewise, incremental rather than immediate induction of hypertension could have been considered, as it has been reported to be associated with a reduced rate of complications when compared to an immediate induction protocol (Veldeman et al., 2022). Thus, given that several of the physiological changes associated with pregnancy may increase the risk for hemodynamic disturbances during treatment of DCI (see below), incremental induction of hypertension under close neuromonitoring could help to further optimize the risk-benefit balance in patients with aSAH during pregnancy.

In general however, DCI remains a common complication after aSAH that affects about one third of the patients who survive the initial bleed, is difficult to treat and often associated with death or significant long-term impairments (Geraghty and Testai, 2017). Moreover, even though DCI and DCI-related infarction after aSAH have traditionally been attributed to vasoconstriction of large caliber vessels, there is increasing evidence that microvascular dysfunction, neuroinflammation and other factors play a role as well and that resolution of angiographic vasospasm is not necessarily associated with a resolution of DCI (Geraghty and Testai, 2017; Naraoka et al., 2014; Suzuki et al., 2021). In any case, it is interesting to note that none of the reviewed case reports described angiographic vasospasm as a complication of aSAH during pregnancy, supporting a previous notion that physiologic hypervolemia and hemodilution during pregnancy may afford some degree of protection against cerebral vasospasm after aSAH (Stoodley et al., 1998). On the other hand, while hypervolemia and hemodilution, especially when combined with induced hypertension (e.g. triple H therapy), can be effective for prevention and treatment for cerebral vasospasm, they are also associated with an increased risk of hemodynamic disturbances including cardiopulmonary failure (Adamczyk et al., 2013; Fennell and Levy, 2019) and may thus have contributed to the poor outcome in our patient. In addition, there are reports that the hormonal and nervous system changes associated with pregnancy could predispose pregnant woman to refractory forms of cerebral and coronary vasospasm (Ergle and Bernard, 2019; Çöven et al., 2017). As such, a better understanding of the mechanisms underlying these complications as well as their relationship to the physiological changes associated with pregnancy will be required before firm conclusions regarding the risk of cerebral vasospasm and other complications after aSAH during pregnancy can be drawn.

## 5 Conclusion

In conclusion, while there are still no formal studies that could guide the optimal management of aSAH during pregnancy, prompt treatment of the ruptured aneurysm and continuation of the pregnancy seems to be associated with a good outcome for both, mother and child in the majority of patients presenting before the third trimester. In patients presenting with aSAH during the third trimester, development of the child has typically progressed far enough that CS before aneurysm treatment is a feasible option that may simplify subsequent treatment decisions as well as the management of potential complications. Nevertheless, development of an individual and interdisciplinary treatment plan for child and mother remains mandatory in every case of aSAH during pregnancy.

## Ethical approval

At the University Bonn and RWTH Aachen, retrospective studies do not need ethical approval.

Informed consent has been obtained from the patient's family for publication of the case report and accompanying images.

## Declaration of competing interest

The authors declare that they have no known competing financial interests or personal relationships that could have appeared to influence the work reported in this paper.
